# A novel diagnostic model based on lncRNA PTPRE expression, neutrophil count and red blood cell distribution width for diagnosis of seronegative rheumatoid arthritis

**DOI:** 10.1007/s10238-024-01343-x

**Published:** 2024-04-25

**Authors:** Jinfang Xia, Huali Gao, Jifeng Tang, Renquan Jiang, Lianbo Xiao, Huiming Sheng, Jinpiao Lin

**Affiliations:** 1 Department of Laboratory Medicine, Tongren Hospital, Shanghai Jiao Tong University School of Medicine, Shanghai, China; 2 Department of Laboratory Medicine, The First Affiliated Hospital of Fujian Medical University, Fuzhou, China; 3 Department of Orthopedic Surgery, Guanghua Hospital Affiliated to Shanghai University of Traditional Chinese Medicine, Shanghai, China; 4 Institute of Arthritis Research in Integrative Medicine, Shanghai Academy of Traditional Chinese Medicine, Shanghai, China

**Keywords:** Rheumatoid arthritis, Long noncoding RNA, Seronegative RA, Diagnostic model

## Abstract

Diagnosis of seronegative rheumatoid arthritis (SNRA) is difficult due to the lack of diagnostic markers. The study aims to construct a novel diagnostic model based on long noncoding RNAs (lncRNAs) expression and laboratory indicators to provide a new idea for diagnostic methods of SNRA. Differentially expressed lncRNAs in peripheral blood cells of RA patients were screened through eukaryotic long noncoding RNA sequencing and validated by quantitative real-time PCR. Meanwhile, the correlation between lncRNAs expression and laboratory indicators was analyzed. The diagnostic value was evaluated by receiver operating characteristic curve analysis. Finally, combined with laboratory indicators, a diagnostic model for SNRA was constructed based on logistic regression and visualized by nomogram. Expression of ADGRE5, FAM157A, PTPN6 and PTPRE in peripheral blood was significantly increased in RA than healthy donors. Meanwhile, we analyzed the relationship between lncRNAs and erythrocyte sedimentation rate, C-reactive protein and CD4 + T cell-related cytokines and transcription factors. Results showed that FAM157A and PTPN6 were positively related to RORγt, and negatively related to GATA3. Moreover, PTPRE has potential discrimination ability between SNRA and healthy donor (AUC = 0.6709). Finally, we constructed a diagnostic model based on PTPRE, neutrophil count and red blood cell distribution width (RDW). The AUC of the model was 0.939 and well-fitted calibration curves. Decision curve analysis indicated the model had better predict performance in SNRA diagnosis. Our study constructed a novel diagnostic model based on PTPRE, neutrophil count and RDW which may serve as a potential tool for the diagnosis of SNRA.

**Supplementary Information:**

The online version contains supplementary material available at 10.1007/s10238-024-01343-x.

## Introduction

Rheumatoid arthritis (RA) is a common chronic autoimmune disease that can affect multiple joints [1, 2]. It is characterized by symmetrical joint pain, swelling and stiffness accompanied by progressive joint destruction and disability [3]. The pathogenesis of RA is still not fully elucidated, and the existing treatments are not yet able to completely cure RA but only control inflammation and delay progression [4]. Therefore, timely and accurate diagnosis and treatment can reduce irreversible joint injury and disability in RA patients [5, 6], which is of great significance for the survival and prognosis of RA patients.

Currently, the clinical diagnosis of RA is mainly based on the patient’s clinical symptoms, X-ray findings and classical laboratory indicators. However, at the early stage, the clinical representations of RA are relatively diverse. The traditional laboratory diagnostic indicators have many limitations in clinical practice, which are prone to missed diagnosis and misdiagnosis, thus causing patients to miss the best opportunity for treatment. The classification criteria of RA are based on the 2010 American College of Rheumatology/European League Against Rheumatism (ACR/EULAR) classification criteria [7]. The specific biomarkers for the detection of RA provided in the 2010 ACR criteria are anti-cyclic citrullinated peptide antibodies (anti-CCP) and rheumatoid factor (RF) [8, 9]. Clinically, RA patients can be divided into serologically positive (SP) RA patients (RF+ and/or anti-CCP+) and serologically negative (SN) RA patients (RF− and anti-CCP−) based on these two indicators. Moreover, when anti-CCP and RF are both negative, more than ten joints must be affected to be considered RA according to the 2010 ACR criteria. Therefore, it is urgent to find potential diagnostic tools for SNRA patients to improve the accuracy of diagnosis. This will significantly reduce the missed diagnosis rate and misdiagnosis rate of RA, which is of great significance for the timely clinical diagnosis and prognosis of RA patients.

In our previous studies, we focus on the role of transcription factor YY1, Th17 cell differentiation, inflammatory factor IL-6, matrix protein Cyr61, red blood cell distribution width, etc., in RA pathogenesis [10–13] and the laboratory diagnosis of RA. Previous studies have indicated [14] that peripheral blood circulating microRNAs miR-22-3p and let-7a-5p have high diagnostic potential in RA. We realized that molecular diagnostic markers may be a good alternative or complementary for traditional serological diagnostic markers. Therefore, the diagnostic value of molecular biomarkers in RA has received much attention.

Long noncoding RNA (LncRNA) is longer than 200 nucleotides noncoding RNAs [15] and cannot encode proteins [16–18], and it is well known that lncRNAs regulate gene expression mainly through various interactions with DNA, RNA and proteins [19, 20]. Therefore, lncRNAs are involved in various critical regulatory processes, such as X-chromosome silencing, chromatin modification, transcriptional activation interference and post-transcriptional modification [21]. In addition, lncRNAs are widely distributed in a variety of bodily fluids and have been shown to be quite stable in plasma, which may serve as biomarkers for various diseases [22]. Meanwhile, from detection perspective, a single quantitative real-time PCR (qRT-PCR) could simultaneously detect multiple lncRNAs. These studies support the innate advantages of lncRNAs in establishing a combined diagnosis of multiple indicators. In clinical studies, researchers have found that lncRNAs are independent risk factors for a variety of diseases [23, 24], which suggests that lncRNAs have the enormous potential to replace or supplement conventional diagnostic markers. It has been found that circulating lncRNASNHG11 in peripheral blood can distinguish between precancerous lesions and early tumors of colorectal cancer. The combination of lncRNAs ZFAS1, SNHG11, LINC00909 and LINC00654 showed a good diagnostic effect in the colorectal cancer (AUC = 0.937) [25]. However, compared with other diseases, there are few studies on lncRNAs as biological diagnostic markers for RA. Therefore, a more in-depth study is necessary. Studies have demonstrated that lncRNAs have been shown to be implicated in disease progression of RA, and a variety of lncRNAs are abnormally expressed in synovial cells [26], peripheral blood mononuclear cells and T cells [27–29], which provides theoretical support for lncRNAs as diagnostic markers of RA.

At present, a single assessment indicator often fails to meet current clinical needs, and the development and construction of a multi-indicator combined diagnostic model has gradually become a new trend in disease diagnosis research [30, 31]. The multi-index combined diagnostic model can diagnose or predict certain diseases through multiple clinical indicators or characteristics and provide clinicians with more accurate and reliable clinical diagnosis tools. Therefore, in this study, based on the expression of lncRNA, clinical data and laboratory indicators of patients were also collected to build a diagnostic model.

In this study, we collected peripheral blood cells from RA patients and healthy donors for eukaryotic long noncoding RNA sequencing, and then, the results of lncRNA sequencing were analyzed by bioinformatics, and verified by quantitative real-time PCR (qRT-PCR). The results indicated that lncRNA PTPRE increased in SNRA patients compared with healthy donors (HD). To improve its diagnostic capability in SNRA, we constructed a SNRA diagnostic model based on PTPRE expression, neutrophil count and RDW after logistic regression analysis.

## Materials and methods

### Study population and blood samples

Peripheral blood samples from five SPRA patients, five SNRA patients and five healthy donors (HD) for eukaryotic long noncoding RNA sequencing, and 62 SPRA patients, 34 SNRA patients, 72 osteoarthritis (OA) patients and 40 HD were enrolled for subsequent verification. All samples were obtained between October 2021 to May 2023 from the First Affiliated Hospital of Fujian Medical University and Tongren Hospital, Shanghai Jiao Tong University School of Medicine (Table 1).

**Table 1:** Clinical characteristics of volunteers with SPRA, SNRA, OA and HD

Characteristics	SPRA	SNRA	OA	HD
Count	67	39	72	45
Sex (F/M)	38/29	28/11	47/25	30/15
Age (years)	52 ± 13.27	48 ± 14.69	52 ± 17.33	49 ± 13.14
RF (IU/ml)	120.0(89.0, 134.0)	< 20	< 20	< 20
Anti-CCP (RU/ml)	111.8(94.0, 135.8)	< 5	< 5	< 5
ESR (mm/h)	45.0(18.0, 66.0)	19.0(10.5, 26.8)	14.0(9.5, 19.5)	11.2(6.5, 15.4)
CRP (mg/l)	9.7(1.5, 17.2)	6.2(4.1, 14.6)	0.7(0.5, 2.5)	0.6(0.5, 1.2)

All values are represented as the mean ± standard deviation or the median (interquartile range) depending on whether the parameters follow a normal distribution

Patients fulfilled the 2010 American College of Rheumatology/European League against Rheumatism classification criteria for RA [7]. The diagnosis of OA matches the 1986 classification criteria of the American College of Rheumatology (ACR) [32]. According to the physical examination results, the healthy persons shall be included in the physical examination without any evidence of disease. All participants with a history of severe cardiovascular, endocrine, hepatic, renal and other chronic inflammatory diseases were excluded.

Laboratory parameters of all patients and healthy donors were measured and analyzed at the laboratory department of the First Affiliated Hospital of Fujian Medical University and Tongren Hospital, Shanghai Jiao Tong University School of Medicine. Serum anti-CCP level was measured using a commercial ELISA kit (EURO-IMMUN, Lübeck, Germany). Serum RF and CRP were detected by immunoturbidimetric assay (Dade Behring, Marburg, Germany). ESR was determined using Westergren’s method. While blood routine indexes including RDW and blood cell count were tested using a Siemens ADVIA 2120i analyzer (Siemens Healthcare Diagnostics, Germany). The detection methods and equipments for above indicators and the quality control methods were the same in the two hospitals.

This study was approved by the Institutional Medical Ethics Review Board of the First Affiliated Hospital of Fujian Medical University, Fuzhou, China (MTCA, ECFAH of FMU [2015]084-1) and Tongren Hospital, Shanghai Jiao Tong University School of Medicine, Shanghai, China (AF/SC-13/01.0). Verbal and written informed consent were obtained from all participants. Two ml of peripheral blood was collected with EDTA anticoagulant. The samples were frozen at − 80 °C in TRIzol for preservation.

### RNA isolation and quantitative real-time PCR

Total RNA from human peripheral blood cells was extracted using the TRIzol Reagent. Reverse transcription was performed using random primers and M-MLV RT (Promega) following the manufacturer’s protocol. LncRNAs sequence information comes from the Ensemble database (https://grch37.ensembl.org/index.html), using the national center for biotechnology information (NCBI) online tool to design primers and blasts.

The qRT-PCR reactions were executed using 2 × Taq Pro Universal SYBR qRT-PCR Master Mix (Vazyme) and carried out on a QuantStudio DX (Applied Biosystems Inc., Foster City, USA) according to the manufacturer’s protocol as follows: 95℃ for 30 s, followed by 40 cycles at 95℃ for 10 s, and at 60℃ for 30 s. The relative expression levels of lncRNAs were calculated using the 2^−∆∆Ct^ method. The above primer sequences are listed in supplementary table S1.

### Nomogram construction and validation

Univariate logistic regression analysis screened out the candidate variable, and then, multivariate logistic regression analysis was used to analyze the independent risk factors. A diagnostic model was constructed based on logistic regression and visualized by nomogram using *R *software version 4.1.3. Each variable is distributed on the nomogram according to its weight to get different lines, and the points of each variable correspond to a point. The corresponding “total points” can be obtained by adding the scores associated with each variable. The projection of “total points” can be used to estimate the probability of a correct diagnosis of SNRA.

To evaluate the performance of the nomogram, we used internal validation via a bootstrap method with 1000 re-samples. ROC and calibration curve were performed to evaluate the accuracy of nomogram models, and then, decision curve analysis (DCA) was used to assess the clinical potential application value of the nomogram.

### Statistical analysis

Bioinformatics analysis of sequencing data was performed using R programming language. All statistical differences, ROC curve and correlation analysis were performed using GraphPad Prism 8 statistical software (GraphPad Software Inc., San Diego, USA).

For two groups of comparison, Student’s t test or Wilcoxon rank-sum test with false discovery rate (FDR) correction for multiple comparisons (FDR < 0.01) was used dependent on whether data conformed to a normal distribution. All values were represented as the mean ± standard Similarly, one-way analysis of variance (ANOVA) or Kruskal–Wallis test followed by Tukey’s or Dunn’s multiple comparisons test was performed for comparisons between more than two groups, while the relationships between clinical parameters and lncRNAs were verified using Spearman correlation.

Deviation or the median (interquartile range) depends on whether the parameters follow a normal distribution. All tests were two-tailed, and the *p* value < 0.05 were considered statistically significant (ns, not significant; *p < 0.05; **p < 0.01; ***p < 0.001; ****p < 0.0001).

## Results

### The visual flowchart of this study

**Fig. 1:**
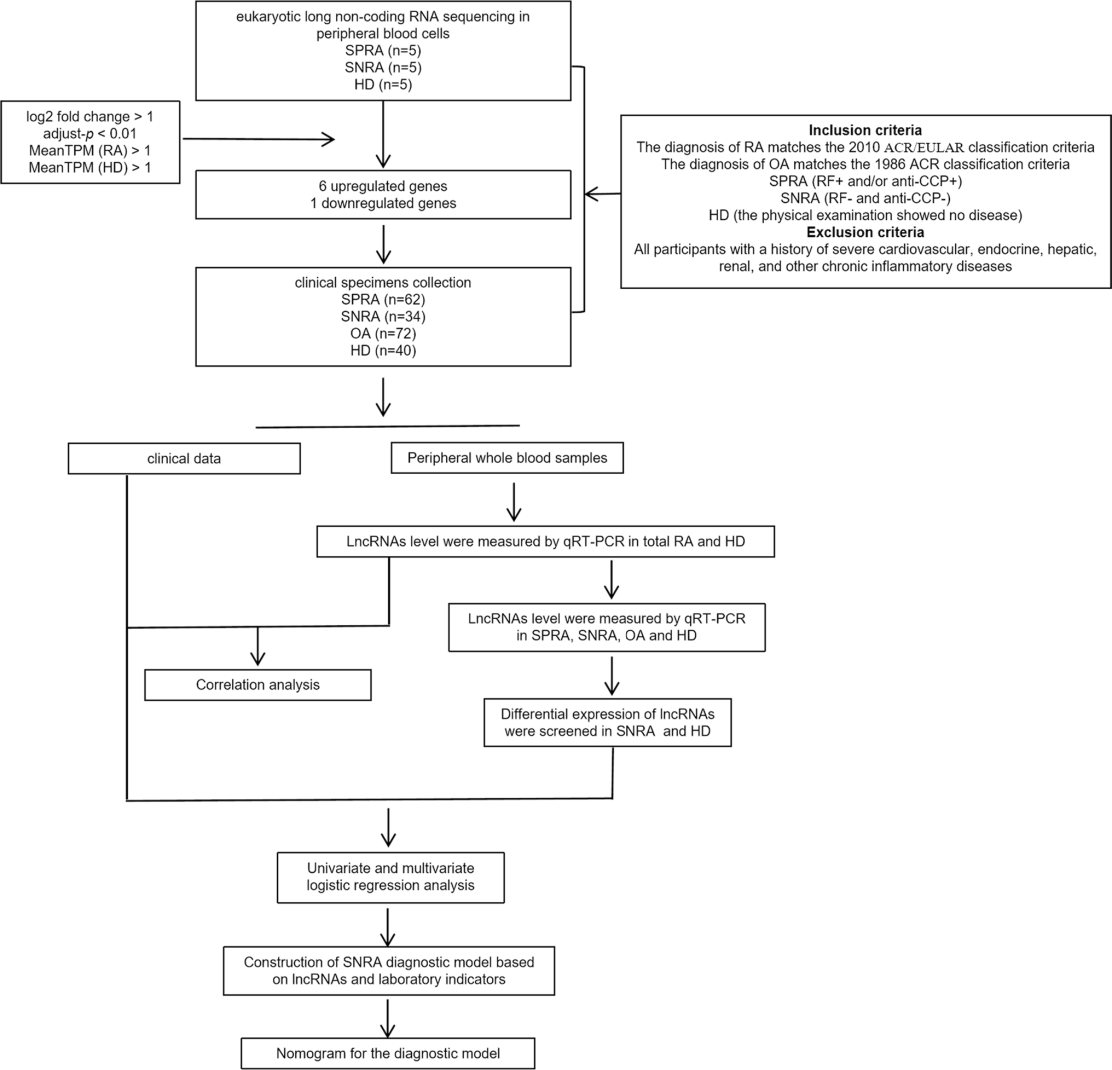
The visual flowchart of this study

Figure 1 presents the overall design of the research. Firstly, we collected peripheral blood cells from five SPRA patients, five SNRA patients and five HD for eukaryotic long noncoding RNA sequencing. Bioinformatics analysis was performed by setting the cutoff value as log2 fold change > 1, adjust-*p* < 0.01, MeanTPM (RA) > 1, MeanTPM (HD) > 1. Six upregulated lncRNAs and one downregulated lncRNA were screened in total RA compared with HD. Then, we collected clinical data and peripheral whole blood samples from 62 SPRA patients, 34 SNRA patients, 72 OA patients and 40 HD to verify the expression of seven lncRNAs by qRT-PCR. Subsequently, the correlation between differentially expressed lncRNAs in total RA and laboratory indicators was analyzed. Moreover, we further divided RA patients into SNRA group and SPRA group to screen out the differentially expressed lncRNAs in SNRA. To improve the diagnostic capability of PTPRE in SNRA and HD, we constructed a SNRA diagnostic model based on PTPRE expression and laboratory inflammatory indicators after univariate and multivariate logistic regression analysis, and visualized by nomogram.

### LncRNAs ADGRE5, FAM157A, PTPN6 and PTPRE were upregulated in RA patients

**Fig. 2:**
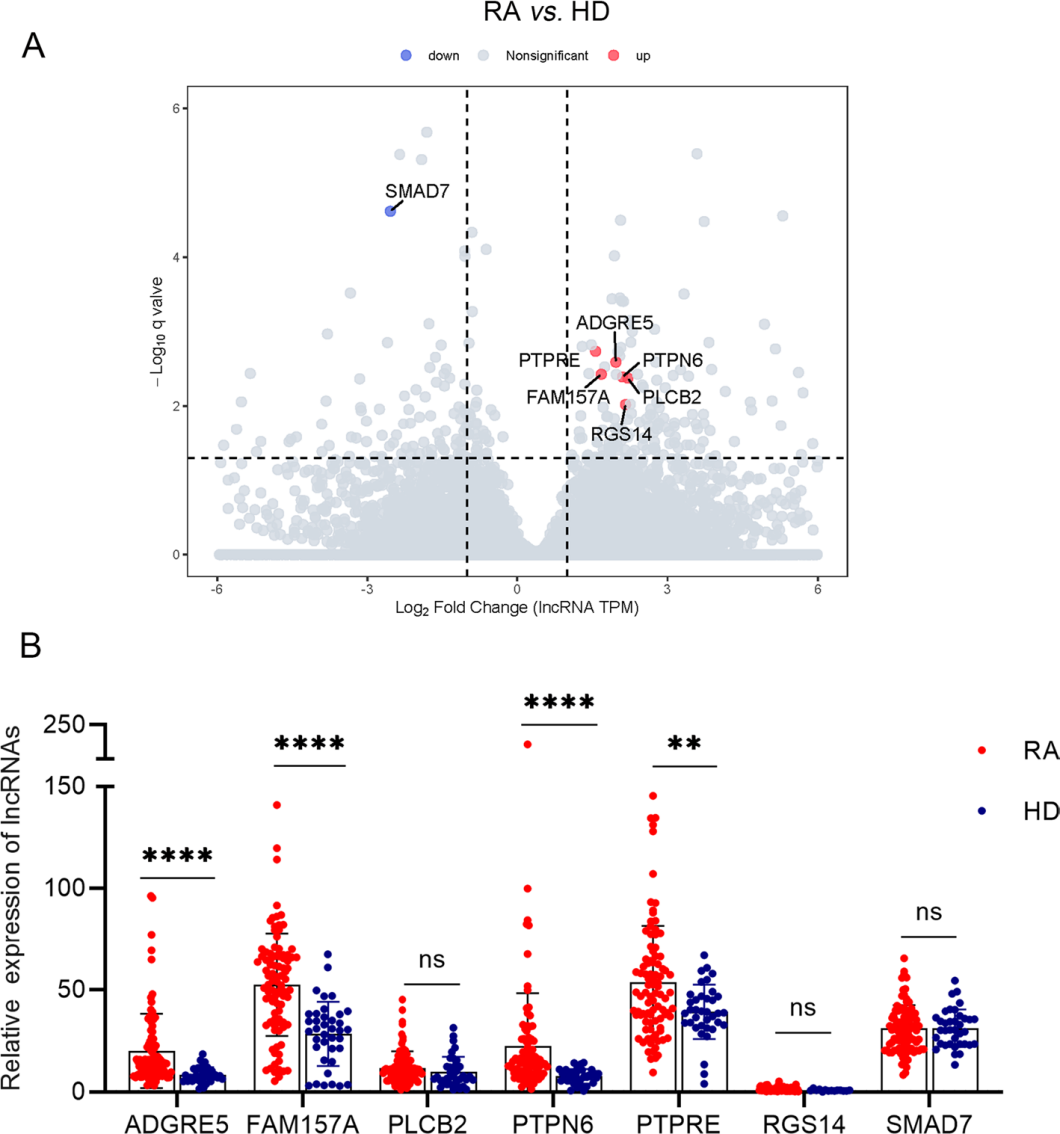
Differential expression of lncRNAs in RA patients and HD. **a** Volcano map of long noncoding RNA sequencing results in RA versus HD. **b** The relative expression of seven candidate lncRNAs in peripheral blood cells of patients with RA (*n* = 96) and HD (*n* = 40). All data presented as mean ± SD, *p < 0.05, **p < 0.01, ***p < 0.001, ****p < 0.0001, ns = not significant

To obtain potentially different lncRNAs in RA patients and healthy donors, we collected peripheral blood cells from 10 RA patients and five healthy donors for eukaryotic long noncoding RNA sequencing. In order to show the differentially expressed genes more vividly, we drew the volcano plot (Fig. 2a), which highlighted in red/blue points (upregulated/downregulated) with log2 fold change > 1, adjust-*p* < 0.01, MeanTPM (RA) > 1, MeanTPM (HD) > 1, including ADGRE5, FAM157A, PLCB2, PTPN6, PTPRE, RGS14 and SAM7 (supplementary table S2).

We collected peripheral blood cells of 96 RA patients and 40 HD to verify the expression of 7 lncRNAs. The qRT-PCR results demonstrated that expressions of ADGRE5, FAM157A, PTPN6 and PTPRE were significantly higher in RA patients compared to HD (Fig. 2b).

### LncRNAs ADGRE5, FAM157A, PTPN6 and PTPRE expression were not correlated with CRP and ESR

**Fig. 3:**
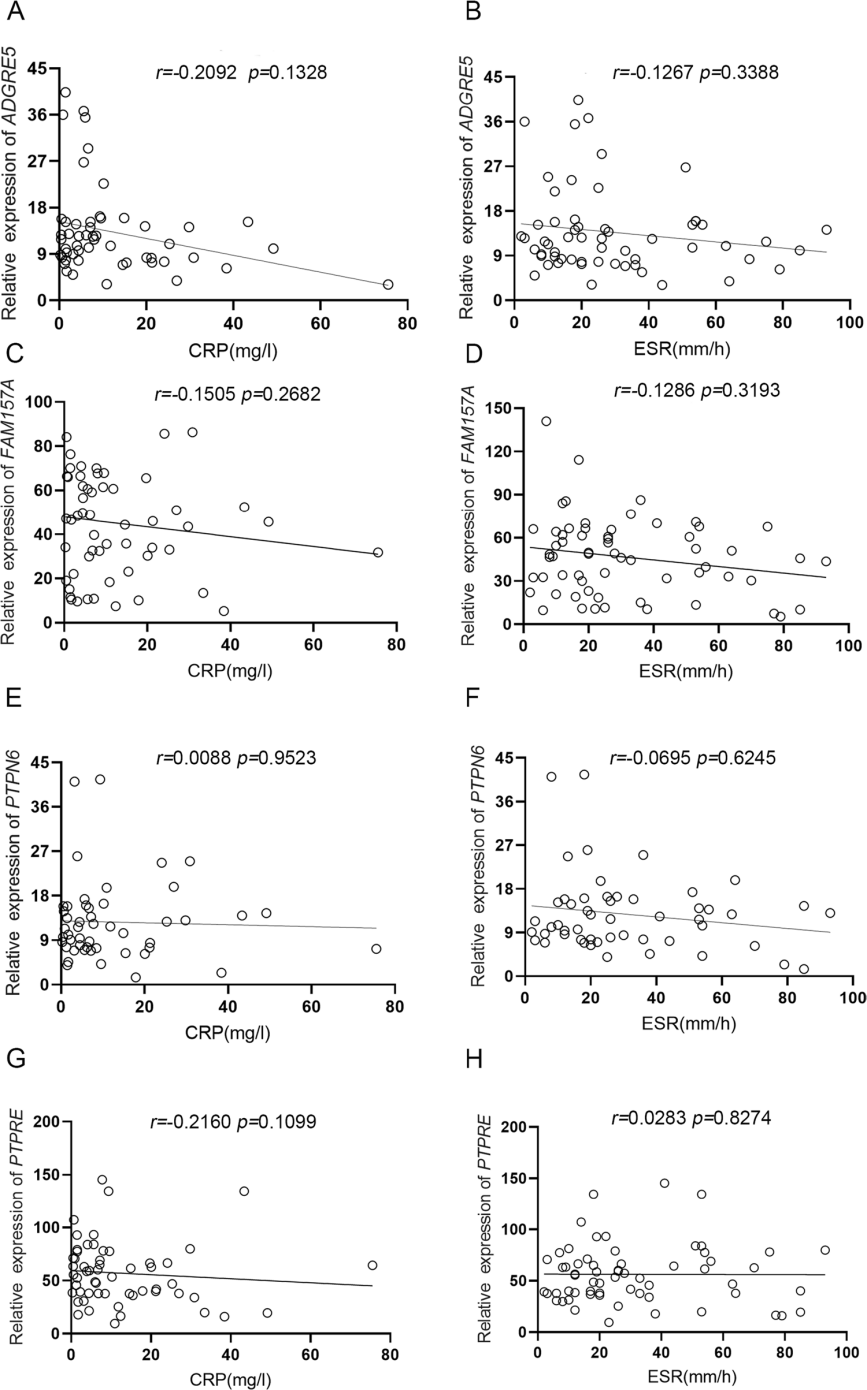
Correlation analysis between lncRNAs expression and RA disease activity. **a**, **c**, **e**, **g** Relationship between lncRNAs expression and CRP in RA (*n* = 73). **b**, **d**, **f**, **h** Relationship between lncRNAs expression and ESR in RA (*n* = 73)

Erythrocyte sedimentation rate (ESR) and C-reactive protein (CRP) were used as the laboratory measure of disease activity of RA [33]. Therefore, the Spearman correlation was used to analyze the relationship between the expression of RA lncRNAs and disease activity. The results showed that no clear correlation was seen between 4 lncRNAs and CRP, as well as ESR (Fig. 3).

### Correlation analysis between lncRNA expression level and CD4 + T cell-related cytokines and transcription factors

Several studies reported that lncRNAs were differentially expressed in CD4 + T lymphocytes, which play the important roles in RA pathogenicity [29, 34, 35]. Therefore, the expression of CD4 + T cell-related cytokines and transcription factors in RA patients was evaluated at the RNA level by qRT-PCR. Spearman correlation was analyzed and the results showed that ADGRE5 and PTPRE were negatively related to IL-10; FAM157A and PTPN6 were positively related to RORγt, and negatively related to GATA3 (Table 2). The above results suggested that the upregulated expression of these lncRNAs may be associated with inflammatory response in RA and may be associated with CD4 + T cell subsets such as Th2, Th17 and Treg.

**Table 2:** Correlation analysis between lncRNAs expression level and CD4 + T cell-related cytokines and transcription factors

	ADGRE5		FAM157A		PTPN6		PTPRE	
	*r*	*p*	*r*	*p*	*r*	*p*	*r*	*p*
IFN-γ	0.2239	0.1037	0.0607	0.6539	0.3351	0.8111	0.1171	0.3856
T-bet	− 0.2582	0.0674	0.0938	0.5082	0.1982	0.1633	− 0.2542	0.0718
IL-4	− 0.1427	0.2986	0.3009	0.0229*	0.3696	0.0055**	− 0.0158	0.9060
GATA3	− 0.1462	0.3058	− 0.3832	0.0051**	− 0.4155	0.0024**	− 0.0556	0.6955
IL-17	0.0592	0.6799	0.2537	0.0667	0.2937	0.0346*	− 0.1490	0.2965
RORγt	− 0.2847	0.0351*	0.3988	0.0019**	0.4465	0.0006***	− 0.2169	0.1084
IL-10	− 0.2953	0.0286*	0.3544	0.0063**	0.3666	0.0054**	− 0.2756	0.0363*
Foxp3	− 0.2627	0.0504	0.3542	0.0064**	0.2312	0.0865	− 0.1469	0.2668

Th1 main cytokine and transcription factor (IFN-γ and T-bet). Th2 main cytokine and transcription factor (IL-4 and GATA3). Th17 main cytokine and transcription factor (IL-17 and RORγt). Treg main cytokine and transcription factor (IL-10 and Foxp3). The asterisk indicates statistically significant results. *p < 0.05, **p < 0.01, ***p < 0.001, ****p < 0.0001

### LncRNA PTPRE was upregulated in SNRA patients

**Fig. 4:**
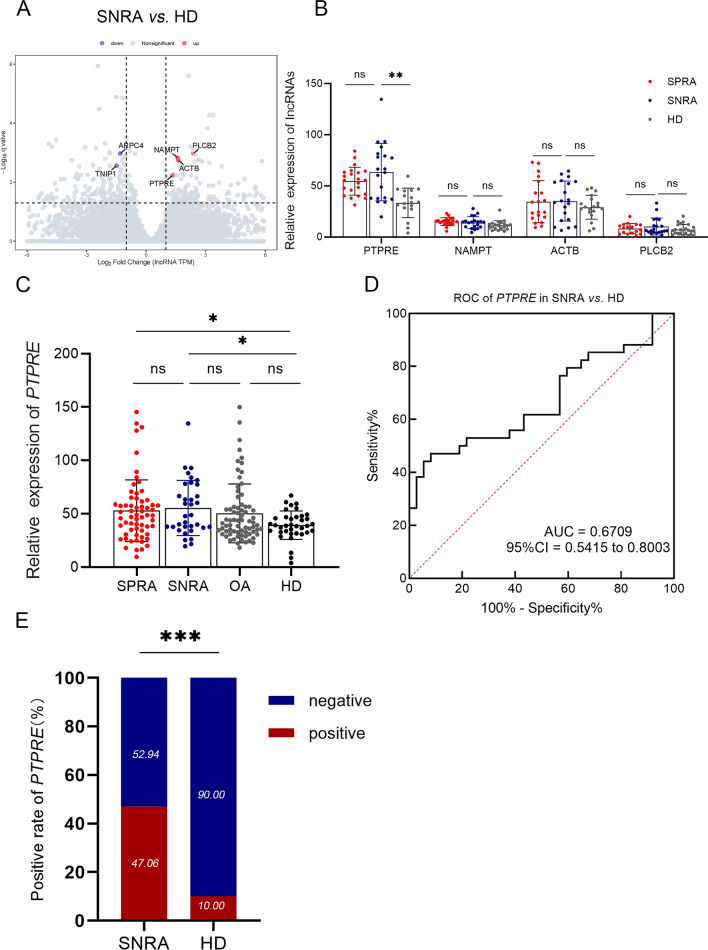
The diagnostic value of lncRNAs in SNRA and HD. **a** Volcano map of long noncoding RNA sequencing results in SNRA versus HD. **b** The relative expression of four candidate lncRNAs in peripheral blood cells of patients with SPRA, SNRA and HD. **c** The expression of lncRNA PTPRE in different groups. **d** ROC curve of lncRNA PTPRE between SNRA and HD. **e** The positive rates of lncRNA PTPRE in SNRA and HD

In the preceding experiments, we found that ADGRE5, FAM157A, PTPN6 and PTPRE were upregulated in RA patients. Since the lack of specific diagnostic markers for SNRA, we further divided RA patients into SNRA group and SPRA group. The results showed that PTPRE, NAMPT, ACTB and PLCB2 were upregulated in SNRA patients than that in HD (supplementary table S2, Fig. 4a). It was further verified by qRT-PCR, results showed only PTPRE had a significantly higher expression in SNRA (*p* < 0.05) compared with HD, and no difference was found in SPRA and SNRA subgroup (Fig. 4b). Moreover, we compared the expression of PTPRE between SPRA, SNRA, OA and HD groups, and no difference was found in OA and HD (Fig. 4c), which indicated that PTPRE may be a specific marker for SNRA diagnosis. The diagnostic value of PTPRE was then evaluated by using receiver operating characteristic (ROC) curve analysis (AUC = 0.6709). We classified SNRA patients as PTPRE-positive or PTPRE-negative based on the optimal positive cutoff value of ROC curve (> 58.073). 47.06% (16/34) was found to be positive for PTPRE in SNRA patients (Fig. 4d, e).

### Nomogram was constructed based on PTPRE, neutrophil count and RDW for SNRA

Laboratory indicators associated with RA progression were selected on the basis of PTPRE. Univariate and multivariate logistic regression analysis was performed to screen the independent risk factors with *p* < 0.05. The clinical variables under statistical analysis were as follows Table 3.

**Table 3:** Univariate and multivariate logistic regression analysis of variables

Variable	Univariable analysis		Multivariable analysis	
	OR (95% CI)	*p*	OR (95% CI)	*p*
PTPRE	1.05(1.02–1.08)	0.0031	1.05 (1.01–1.10)	0.039
NEUT	2.02(1.35–3.02)	0.0007	1.88 (1.05–3.37)	0.034
MONO	1.84(1.23–2.73)	0.0028		
LYMPH	1.40(0.67–2.95)	0.3726		
HGB	0.96(0.93–0.99)	0.0211		
RDW	5.62(2.29–13.78)	0.0002	3.39 (1.46–7.88)	0.005
PLT	1.02(1.01–1.02)	0.0010		
MPV	0.38(0.21–0.70)	0.0018		
PDW	0.87(0.79–0.96)	0.0069		

*NEUT* neutrophil count, *MONO* monocyte count, *LYMPH* lymphocyte count, *HGB* hemoglobin, *RDW* red blood cell distribution width, *PLT* platelet count, *MPV* mean platelet volume, *PDW* platelet distribution width, *OR* odd ratio

**Fig. 5:**
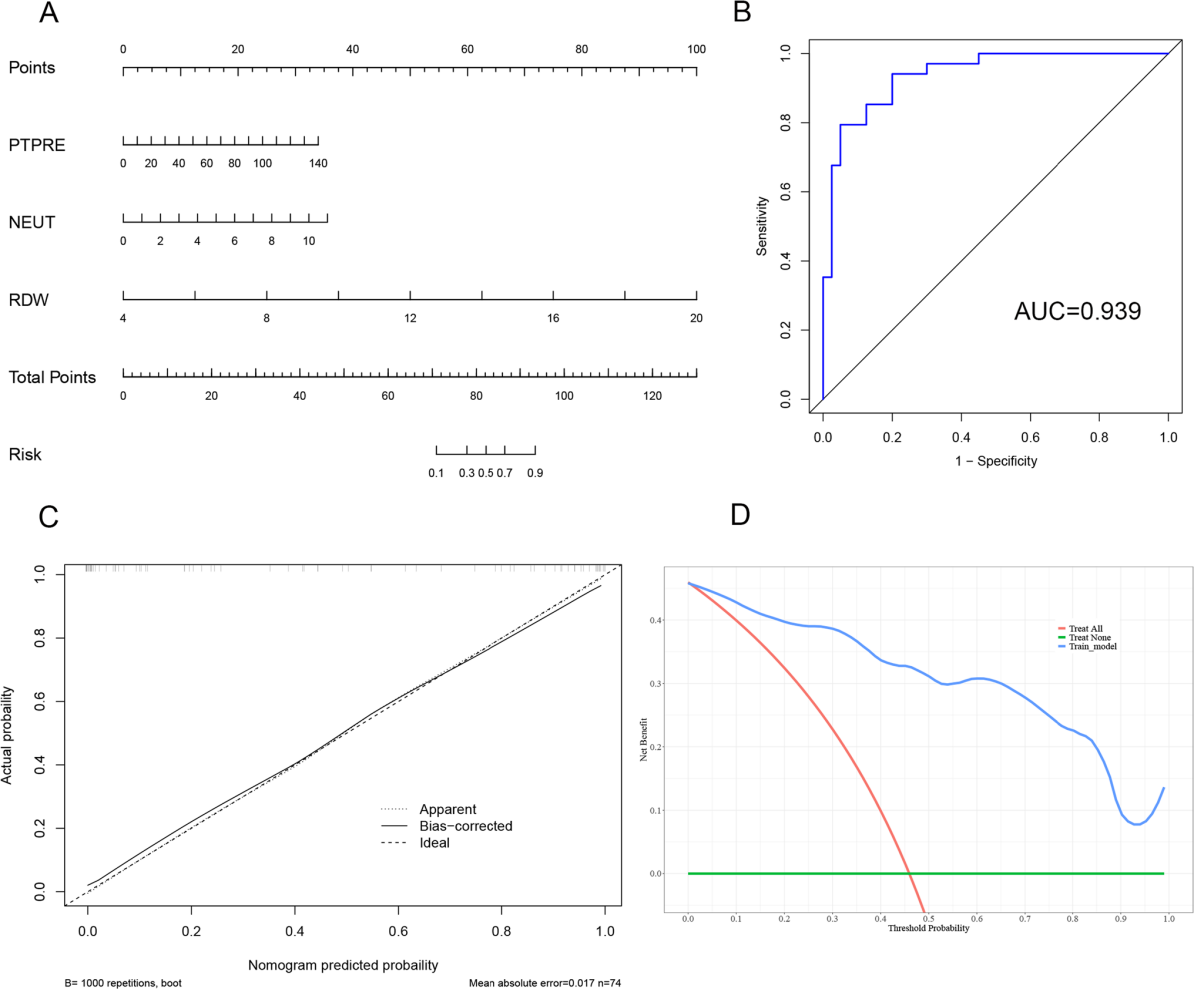
Construction and verification of nomogram. **a** Nomogram to evaluate the probability of a correct diagnosis of SNRA. **b** ROC curve of the nomogram model. **c** Calibration curve of the nomogram model. **d** DCA curve of the nomogram model. *NEUT* neutrophil count, *RDW* red blood cell distribution width

According to the results of univariate and multivariate regression analysis, a SNRA nomogram was drawn based on the multivariate logistic regression model, including PTPRE, neutrophil count and RDW (Fig. 5a). The final model was validated internally by using the bootstrap method (1000 repetitions). The model showed good performance with AUC of 0.939 and well-fitted calibration curves (Fig. 5b, c). In addition, from 0 to 1 on the abscissa, the blue line in the DCA curve is far from and consistently above the red and green lines, manifesting that decision making based on the nomogram model may benefit SNRA patients (Fig. 5d).

## Discussion

Rheumatoid arthritis is a chronic autoimmune disease that initially presents with joint pain and swelling. However, there are often no obvious subjective symptoms in the early stage. In such instances, serology indicators represent an essential complementary diagnostic tool. Thus, a diagnostic dilemma would occur with the diagnosis of SNRA. RA rapidly progresses to cause joint deterioration and functional disability, eventually leading to unfavorable disease outcomes. Therefore, there is an urgent need to find new diagnostic markers or models for RA patients, especially for SNRA patients. Timely and accurate diagnosis is imperative.

Based on prior studies, we realized that molecular diagnostic markers may be a useful alternative or supplementary for traditional serological diagnostic markers. Therefore, we focus our sights on lncRNAs (one of the molecular biomarkers), which has a highly tissue/cell specific conserved secondary structure and was stably present in body fluids [36]. Secondly, the examination method is simple and convenient, and a single qRT-PCR reaction can simultaneously detect multiple lncRNAs, which has the potential of multi-index combined detection. These evidences above suggest that the potential of lncRNA as a diagnosis biomarker in RA.

In this study, we first analyzed lncRNAs expression in RA and HD by lncRNA sequencing and the upregulated lncRNAs were preliminarily screened through bioinformatics analysis and qRT-PCR validation, including ADGRE5, FAM157A, PTPN6 and PTPRE. In RA patients, ESR and CRP can reflect the state of systemic inflammation and disease activity to a certain extent. Therefore, we performed correlation analysis, and the results indicated no obvious correlation between the four lncRNAs and ESR as well as CRP, which suggested that the abnormal expression of these four lncRNAs may be independent of traditional inflammatory markers.

RA is a chronic inflammatory disease, characterized by intense, destructive infiltration of synovial tissue by a broad spectrum of inflammatory cells. CD4 + T cells constitute a large proportion of the inflammatory cells invading the synovial tissue. Upon antigenic stimulation and cytokine signaling, naive CD4 + T cells activate and differentiate into various T helper cell subsets [37]. Many studies have found that CD4 + T cell signaling abnormalities, cytokines and chemokines production, and T cell differentiation is associated closely with the aberrant expression of several lncRNAs [29, 34, 35]. In this study, it was found that ADGRE5 and PTPRE were negatively related to IL-10 (main cytokine for Treg); FAM157A and PTPN6 were positively related to RORγt (main transcription factor for Th17), and negatively related to GATA3 (main transcription factor for Th2). These results suggested that the upregulated expression of these lncRNAs may be associated with inflammatory response in RA and may be associated with CD4 + T cell subsets; however, the exact correlation and mechanism remain to be further studied.

Diagnosing SNRA can be challenging due to complex diagnostic criteria. We further mined the sequencing data, the results show that PTPRE was upregulated in SNRA patients than that in HD. At present, a single indicator has certain limitations for clinical applications due to the lack of good discriminative ability, which may be affected by many interference factors. This also could cause some bias or misclassification, and failure to predict the disease development, etc. Therefore, the development and construction of multi-indicators combined diagnostic model has gradually become a new trend in disease diagnosis research. Thus, to improve the diagnostic capability of PTPRE in SNRA, we planned to construct a SNRA diagnostic model based on PTPRE expression and laboratory indicators of inflammation which have previously been reported to be associated with RA, including common inflammatory and immune cells (NEUT: neutrophil, MONO: monocytes, LYMPH: lymphocytes), and some inflammatory indicators of blood test (HGB, RDW, PLT, MPV and PDW). According to the results of univariate and multivariate regression analysis, a diagnostic model was constructed based on PTPRE, neutrophil count and RDW for SNRA in this study and presented as a nomogram. The model showed good performance with AUC of 0.939 and well-fitted calibration curves. DCA showed superior overall net benefit. In our previous study, we found that RDW was increased in patients with RA which was associated with inflammation of RA, suggesting that RDW may be a potential auxiliary marker for indicating inflammation process in RA [13]. However, RDW is not a specific indicator of RA, especially in SNRA. As expected, the changes in RDW have a significant impact on the diagnosis of SNRA. Thus, the constructed nomogram includes PTPRE and RDW. The addition of PTPRE enhanced the specificity and discriminatory capacity of diagnostic model. However, the observation indicators were not comprehensive enough. We will continue to delve into the issues in the follow-up study.

## Conclusion

In this study, lncRNA PTPRE is expected to be new biomarker for the differential diagnosis of SNRA and HD. In addition, a diagnostic model based on PTPRE, neutrophil count and RDW provided a novel diagnostic model for SNRA, which has significant clinical application value.

## Supplementary Information

Below is the link to the electronic supplementary material.

Supplementary file1 (DOCX 13 kb)

## Data Availability

The raw data can be obtained on request from the corresponding author.
