# Supplementary material for: A novel diagnostic model based on lncRNA PTPRE expression, neutrophil count and red blood cell distribution width for diagnosis of seronegative rheumatoid arthritis
Source: Clin Exp Med. 2024 Apr 25;24(1):86. doi: 10.1007/s10238-024-01343-x (PMC11045583; doi:10.1007/s10238-024-01343-x)
Supplement: Supplementary file 1 — Supplementary file1 (DOCX 13 kb) [file 10238_2024_1343_MOESM1_ESM.docx]

**Supplementary materials and methods**

Supplementary Table S1

Primers used for qRT-PCR.

| Species | lncRNA Name | Sequence (5′-3′) |
| --- | --- | --- |
| Homo sapiens | ACTB | Fw: CCTGAAGTACCCCATCGAGC |
|  |  | Rv: TCTGCAGAGTTCCAAAGGAGAC |
|  | ADGRE5 | Fw: AGACAGGCAGCTAGTTCTCC |
|  |  | Rv: GTGGCTGCACCCAGTGTAT |
|  | FAM157A | Fw: CCAGATTTGCAGTGTCCCAC |
|  |  | Rv: CCCAAGTGTGGCATGAGGTA |
|  | PLCB2 | Fw: GTGCCCATGACACGAAGTCT |
|  |  | Rv: GTAGGTGTGCGGACCGAAG |
|  | PTPN6 | Fw:AACTTCCTACTGCACTGCTCC |
|  |  | Rw:TCAGCATGGCTTCACTTCCC |
|  | PTPRE | Fw:GTGGGTATCACTGTGCCGAG |
|  |  | Rw:TCCAAGATTCCGTTGGGCAT |
|  | RGS14 | Fw:TCGCGTTTGTCCTGGGAAGG |
|  |  | Rw:TGGCTGAGATTCGTACCACG |
|  | NAMPT | Fw:TGCTGGCATAGGAGCATCTG |
|  |  | Rw:TCATGGTCTTTCCCCCAAGC |
|  | SMAD7 | Fw:TCTCCCCACCCCCAAATTAAG |
|  |  | Rw:TTCCTACATGGAAGGTCCGC |

Fw: Forward primer; Rv: Reverse primer;

Supplementary Table S2
Information about candidate lncRNAs.

| Entrez Gene ID | GeneName | Gene Description |
| --- | --- | --- |
| ENST00000484841 | ACTB | actin beta |
| ENST00000591737 | ADGRE5 | adhesion G protein-coupled receptor E5 |
| ENST00000437428 | FAM157A | family with sequence similarity 157 member A |
| ENST00000558588 | PLCB2 | phospholipase C beta 2 |
| ENST00000542761 | PTPN6 | protein tyrosine phosphatase, non-receptor type 6 |
| ENST00000487428 | PTPRE | protein tyrosine phosphatase, receptor type E |
| ENST00000514102 | RGS14 | regulator of G protein signaling 14 |
| ENST00000484527 | NAMPT | nicotinamide phosphoribosyltransferase |
| ENST00000545051 | SMAD7 | SMAD family member 7 |
